# Effects of Surface-Bound Collagen-Mimetic Peptides on Macrophage Uptake and Immunomodulation

**DOI:** 10.3389/fbioe.2020.00747

**Published:** 2020-07-03

**Authors:** Andrew T. Rowley, Vijaykumar S. Meli, Natalie J. Wu-Woods, Esther Y. Chen, Wendy F. Liu, Szu-Wen Wang

**Affiliations:** ^1^Department of Chemical and Biomolecular Engineering, University of California, Irvine, Irvine, CA, United States; ^2^Department of Biomedical Engineering, University of California, Irvine, Irvine, CA, United States; ^3^Department of Materials Science and Engineering, University of California, Irvine, Irvine, CA, United States; ^4^The Edwards Lifesciences Center for Advanced Cardiovascular Technology, University of California, Irvine, Irvine, CA, United States

**Keywords:** immunomodulatory material, phagocytosis, cell uptake, microparticle, nanoparticle, macrophage, LAIR-1, collagen peptide

## Abstract

The interaction between collagen/collagen-like peptides and the commonly expressed immune cell receptor LAIR-1 (leukocyte-associated immunoglobulin-like receptor-1) regulates and directs immune responses throughout the body. Understanding and designing these interactions within the context of biomaterials could advance the development of materials used in medical applications. In this study, we investigate the immunomodulatory effects of biomaterials engineered to display a human collagen III-derived ligand peptide (LAIR1-LP) that targets LAIR-1. Specifically, we examine the effects of LAIR1-LP functionalized surfaces on uptake of polymeric particles and cell debris by macrophages polarized toward inflammatory or healing phenotypes. We observed that culture of macrophages on LAIR1-LP functionalized surfaces increased their uptake of PLGA micro- and nano-particles, as well as apoptotic fibroblasts, while reducing their secretion of TNFα in response to LPS/IFNγ pro-inflammatory stimulation, when compared to cells seeded on control surfaces. To investigate the role of LAIR-1 in the observed LAIR1-LP-induced effects, we used siRNA to knock down LAIR-1 expression and found that cells lacking LAIR-1 exhibited enhanced particle uptake on LAIR1-LP and control surfaces. Furthermore, analysis of gene expression showed that LAIR-1 knockdown led to increase expression of other receptors involved in cell uptake, including CD-36, SRA-1, and beta-2 integrin. Together, our study suggests that LAIR1-LP enhances macrophage uptake potentially through interactions with collagen-domain binding surface receptors, and inhibits inflammation through interaction with LAIR-1.

## Introduction

The extracellular matrix protein collagen plays a complex multifaceted role in regulating cells in the immune system and maintaining homeostasis. It is the most abundant protein in the human body and comes in 29 different varieties, all containing the conserved triple helix suprastructure stabilized by repeated amino acid sequences of glycine-X–Y, where X and Y can be any amino acid but are often proline and hydroxyproline, respectively (Gelse et al., 2003). Specific regions of collagen are recognized as ligands by different receptors expressed by immune cells; these receptors include integrins, discoidin domain receptors, immunoglobulin-like receptors, and mannose receptors, resulting in diverse immunomodulatory responses (Leitinger, 2011). Interaction with natural collagen matrices, reconstituted collagen hydrogels, and/or collagen peptides has been shown to increase cellular adhesion and integrin expression in both adaptive and innate immune cells, as well as actively modulate the phenotype of immune cells in a variety of ways, affecting a range of immune cell functions (Reyes and García, 2004; Zaveri et al., 2014; Boraschi-Diaz et al., 2017; Rowley et al., 2019). More specifically, adhesion to collagen generally reduces the inflammatory response of macrophages, and is thought to have a critical regulatory role in setting the threshold for innate immune responses in tissue (Meyaard, 2010). These immune inhibitory functions are in part from its interaction with the commonly expressed leukocyte-associated immunoglobulin-like receptor 1 (LAIR-1 or CD305).

LAIR-1 is a transmembrane glycoprotein with a cytoplasmic tail containing two immune receptor tyrosine-based inhibitory motifs and is expressed in both mice and humans on a majority of immune cell types, including natural killer cells, T cells, B cells, monocytes/macrophages, dendritic cells, eosinophils, basophils, and mast cells (Meyaard, 2008). Upon LAIR-1 binding and activation, the cytoplasmic tail recruits phosphatases SHP-1 and SHP-2, which antagonize activation signals (Daeron et al., 2008; Meyaard, 2008). It has been shown that the binding of LAIR-1 inhibits the activation of immune cells including, T-cells, B-cells, NK cells, dendritic cells and monocytes/macrophages, although interestingly there is also evidence showing LAIR-1 is required in activation of Th17 T cell response (Meyaard et al., 1997; Lebbink et al., 2006, 2009; Meyaard, 2008; Zhang et al., 2014; Agashe et al., 2018). LAIR-1 recognizes ligands that, in part, contain the triple-helical regions of collagen-like domains and are characterized by the repeating glycine-proline-hydroxyproline sequences but not glycine–proline–proline sequences (Lebbink et al., 2009; Meyaard, 2010). The mechanism of LAIR-1 binding to human collagen III is likely conserved in human and mouse. Structure and mutagenesis studies have shown that the amino acids of the LAIR-1 receptor that bind collagen (Arg59, Glu61, and Trp109) are conserved in over 9 different species, including the human and mouse homologs (Brondijk et al., 2010), and translates to nearly identical binding affinities with human collagen III (Lebbink et al., 2006, 2007). The unique role of LAIR-1 and collagen in immune regulation is thought to influence multiple physiological processes including inflammation, wound healing, as well as progression of cancer and other disease states (Meyaard, 2008, 2010; Steevels et al., 2010; Rygiel et al., 2011).

Given its expression on most immune cells, and its inhibitory properties and regulatory role, LAIR-1 is thought to be a potential therapeutic target for diseases including acute myeloid leukemia and other cancers, as well as inflammatory diseases such as fibrosis and arthritis (Kang et al., 2015, 2016; Agashe et al., 2018; Park et al., 2020). For example Kang et al. (2015, 2016) suggested that intervention in LAIR-1 signaling may lead to successful treatment of acute myeloid leukemia, as their data showed LAIR-1 knockdown abolishes leukemia development but does not affect normal hematopoiesis of stem cells. To improve receptor targeting, several studies have identified high LAIR-1 affinity collagen segments (and/or peptides) and characterized their role in T-cell activation and cellular adhesion (Lebbink et al., 2009). A collagen III peptide, referred to here as LAIR-1 ligand peptide (LAIR1-LP), in particular has high affinity for LAIR-1, exemplified by the greatest inhibition of CD3-induced T cell activation (Lebbink et al., 2009). Previously our research group has shown that macrophage interaction with LAIR1-LP functionalized surfaces significantly inhibits LPS-induced TNFα production when compared to cells cultured on control surfaces, for both human and mouse macrophages (Kim et al., 2017).

Since one of the main functions of the innate immune system is to clear foreign particles and cellular debris, the goal of this current study is to investigate the effects of LAIR1-LP functionalized surfaces on macrophage uptake of particles and apoptotic cells. The natural uptake targets of macrophages (e.g., bacteria, viruses, and parasites) span micron to nanometer lengths. Furthermore, synthetic microparticles and nanoparticles have been widely explored for *in vivo* drug delivery and tissue engineering applications and are also in the size range that is examined in this study. We assessed the impact of LAIR1-LP binding on uptake by BMDM polarized toward inflammatory and wound healing phenotypes. We also examined uptake of PLGA micro- and nano-particles, which have been widely explored for drug delivery and tissue engineering applications, as well as apoptotic fibroblasts, which would be present within damaged tissue. To probe the role of LAIR-1, we used siRNA to knockdown receptor expression (LAIR-1 KD) and investigated the effects on uptake responses modulated by LAIR1-LP functionalized surfaces and expression of other receptors involved in uptake. Our study suggest that LAIR1-LP enhances macrophage uptake, and induces a complex multifaceted immune response that decreases TNFα secretion by inflamed macrophages and modulates the phenotype/receptor expression of both stimulated and unstimulated macrophages.

## Materials and Methods

### LAIR-1 Ligand Peptide (LAIR1-LP)

LAIR1-LP was purchased from Genemed Synthesis. It has a sequence of C(GPP)_5_GAOGLRGGAGPOGPEGGKGAA GPOGPO(GPP)_5_-NH_2_ (where “O” is hydroxyproline) and has been described in previous publications (Lebbink et al., 2009; Kim et al., 2017). The peptide includes the collagen III synthetic peptide III-30 sequence known to bind LAIR-1, two (GPP)_5_ flanking regions to ensure triple helical conformation, and a N-terminal cysteine for surface conjugation to maleimide. Circular dichroism analysis was performed to confirm triple-helical conformation (see Supplementary Material).

### Functionalization of Well Surfaces With LAIR1-LP

Maleimide activated clear 8-well strip plates (Pierce^TM^) were functionalized with either cysteine or LAIR1-LP. Cysteine or LAIR1-LP was suspended in an endotoxin-free binding buffer (0.1 M sodium phosphate, 0.15 M sodium chloride, 10 mM EDTA; pH of 7.2) to facilitate thiol bonding to maleimide-activated clear 8-well strip plate (Pierce^TM^) surfaces. Tris(2-carboxyethyl)phosphine (TCEP, 20 mM) in HEPES (50 mM) was added to achieve a final concentration of 2 mM (100× molar excess to the peptide) in order to prevent peptide-to-peptide disulfide bond formation. A final functionalization solution was made of 2 μM LAIR1-LP and 2 mM TCEP in binding buffer. The solution was allowed to react at room temperature for 20 min, added to wells at either 0.2 μM or 0.02 μM of LAIR1-LP, along with binding buffer stock to achieve a 100 μL total volume, and then allowed to react in the wells overnight at 4°C. The wells were then washed 3× with sterile PBS. Final amounts of LAIR1-LP reacted were 100 ng and 1 μg per well. All functionalization chemistry was performed under sterile conditions.

A LAIR1-LP peptide biotinylated at the C-terminus was used to quantify the extent of surface functionalization; streptavidin-horseradish peroxidase (HRP) was reacted in 100x molar excess of surface-conjugated LAIR1-LP for 1 h, washed, and incubated with tetramethylbenzidine (TMB), and the absorbance was measured to quantify the LAIR1-LP surface concentration.

### Macrophage Harvest and Culture

All procedures requiring animal tissues were carried out in accordance with protocols approved by the Institutional Animal Care and Use Committee (IACUC) at the University of California, Irvine. Femurs were harvested from C57BL/6J mice (Jackson Laboratory), and bone marrow from each bone was flushed with Dulbecco’s Modified Eagle’s medium (DMEM) supplemented with 10% heat-inactivated fetal bovine serum (FBS) and then centrifuged to pellet cells. The cell pellet was resuspended in ammonium-chloride-potassium (ACK) lysis buffer (Thermo Fisher) to lyse red blood cells, centrifuged, and then resuspended and cultured in D-10 media. D-10 media consists of high-glucose DMEM supplemented with 10% heat-inactivated FBS, 2 mM L-glutamine, 100 U/ml penicillin, 100 μg/ml streptomycin (Thermo Fisher), and 10% conditioned media from CMG 14–12 cell expressing recombinant mouse macrophage colony stimulating factor (M-CSF). Cells were cultured for 7 days to induce differentiation to bone marrow derived macrophages (BMDMs).

### PLGA Microparticle Synthesis

20 mg of PLGA [Resomer RG 504H, Poly(D,L-lactide-co-glycolide)] and 20 μL BODIPY (1 mg/mL) were dissolved in 1 mL DMC (dimethyl chloride). The mixture was vortexed and dropwise precipitated into 30 mL 1% PVA [poly(vinyl alcohol), 87–90% hydrolyzed, average MW 30,000–70,000]. The solution was mixed using a point-arm sonicator at low speed for 5 min. PLGA was pelleted by spinning at 4,200 rpm for 5 min. Particles were then vacuum filtered, washed with Milli-Q water, and then filtered using 1 and 10 μm sieves to achieve particles with 1–10 μm diameter. After pelleting, particles were stored dry at 4°C for use the next day. PLGA MP were characterized via SEM images after being sputter-coated with 5 nm of iridium using a SEM (Quanta3D EDAX-TSL Q3D) at 5 kV. ImageJ analysis was used to quantify average diameter and PDI of the PLGA MP population using SEM images.

### PLGA Microparticle Uptake

**FIGURE 1:**
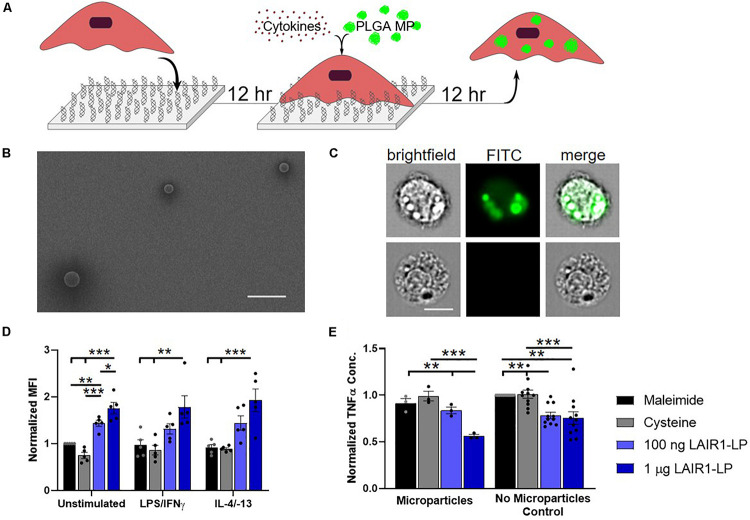
Effects of LAIR1-LP-conjugated surfaces on the uptake of PLGA microparticles (MPs) by BMDMs. **(A)** Diagram depicting experimental conditions and timeline used to investigate LAIR1-LP modulated PLGA MP uptake. **(B)** Representative SEM image of PLGA MPs. Average particle diameter was 2.5 ± 0.75 μm, with a PDI of 0.09 (Scale bar = 10 μm). **(C)** Representative flow cytometry microscopy images are shown of unstimulated BMDMs, cultured on control (maleimide) surfaces and incubated with BODIPY-labeled PLGA MPs. Columns, from left to right, are images in brightfield, FITC wavelength (BODIPY dye), and combined brightfield/BODIPY (Scale bar = 10 μm). **(D)** Mean fluorescence intensity of BODIPY-labeled PLGA MPs, in macrophages with internalized particles. Values have been normalized to the unstimulated BMDMs on maleimide surfaces. **(E)** Pro-inflammatory TNFα concentrations secreted by BMDMs that were stimulated with LPS/IFNγ, on the different surfaces, with and without PLGA MPs. Values were normalized to the TNFα concentration for the maleimide surface with no microparticles (control). For panels **(D,E)**, values reported are average ± SEM, with *N* ≥ 3 independent biological replicates, and the dots are individual data points. Statistical significance was determined by one-way ANOVA with Tukey’s *post hoc* test (*p < 0.05, **p < 0.01, ***p < 0.001).

Bone marrow derived macrophages were seeded in 96-well maleimide surfaces and surfaces functionalized with cysteine or LAIR1-LP as described above for 12 h, and then incubated with stimulation solution and PLGA MPs (150,000 particles/well) for an additional 12 h (Figure 1A). Final concentrations of stimulation solution per well were as follows: 0.08 ng/ml of LPS and 0.26 ng/ml IFNγ in D10 media (inflammatory M1 phenotype), or 2.6 ng/ml IL-4/13 in D10 media (resolution M2 phenotype), or unstimulated groups received only D10 media (naive M0 phenotype). After 12 h of incubation with the particles and cytokines, cell supernatant was removed for cytokine analysis via ELISA, wells were then washed with PBS, and BMDMs were lifted for flow cytometry analysis. Four replicate wells were combined for each condition for each *n*, and the experiment was repeated with cells from multiple mice, *n* ≥ 3.

### PLGA Nanoparticle Synthesis

20 mg of PLGA [Resomer RG 504H, Poly(D,L-lactide-co-glycolide)] and 20 μL BODIPY (1 mg/mL) were dissolved in 1.33 mL of acetone and vortexed. 0.67 mL of methanol was immediately added to the solution and vortexed again. The mixture was dropwise precipitated into 18 mL of 5% Pluronic F-68 in MilliQ water (Gibco Pluronic F-68) with stirring on a stir plate for 2 h covered at room temperature. PLGA was pelleted by spinning at 14,000 rpm for 5 min. Particles were characterized using dynamic light scattering (DLS; Malvern Zetasizer ZS) and stored dry at 4°C for use the next day. PLGA nanoparticles were also imaged and characterized by TEM (JEM-2100F) after being stained with 2% uranyl acetate.

### PLGA Nanoparticle Uptake

**FIGURE 2:**
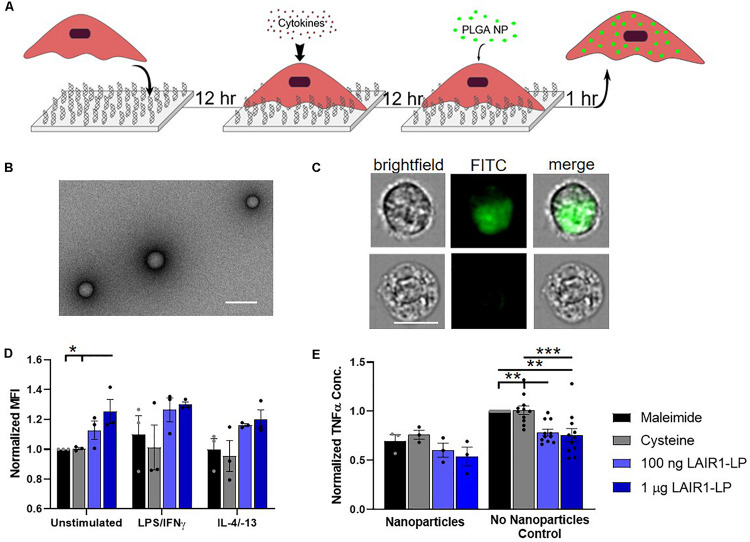
Effects of LAIR1-LP-conjugated surfaces on the uptake of PLGA nanoparticles (NPs) by BMDMs. **(A)** Diagram depicting experimental conditions and timeline used to investigate LAIR1-LP modulated PLGA NP uptake. **(B)** Representative TEM image of PLGA NPs. Average particle diameter was 214 nm with an average PDI of 0.035 by DLS analysis (Scale bar = 500 nm). **(C)** Representative flow cytometry microscopy images are shown of unstimulated BMDMs, grown on maleimide control surfaces and incubated with BODIPY-labeled PLGA NPs. Columns, from left to right, are images in brightfield, FITC wavelength (BODIPY), and combined brightfield/BODIPY (Scale bar = 10 μm). **(D)** Mean fluorescence intensity of BODIPY-labeled PLGA NPs, in macrophages with internalized particles. Values have been normalized to the unstimulated BMDMs on maleimide surfaces. **(E)** Pro-inflammatory TNFα concentrations secreted by BMDMs that were stimulated with LPS/IFNγ, on the different surfaces, with and without PLGA NPs. Values were normalized to TNFα concentration for the maleimide surface with no nanoparticles (control). For panels **(D,E)**, values reported are average ± S.E.M. with *N* ≥ 3 independent biological replicates, and the dots are individual data points. Statistical significance was determined by one-way ANOVA with Tukey’s *post hoc* test (*p < 0.05, **p < 0.01, ***p < 0.001).

Bone marrow derived macrophages were seeded in 96-well maleimide plates on various functionalized surfaces (maleimide, cysteine, and 100 ng or 1 μg LAIR1-LP surfaces) at ∼30,000 cells/well for 12 h (Figure 2A). Due to the rapid uptake of nanoparticles, co-stimulation with cytokines and nanoparticles was not possible. In order to assess the effects of LAIR1-LP on nanoparticle uptake of the various stimulated phenotypes, a pre-stimulation timeline was used. Cells were incubated with cytokines at the concentrations previously mentioned above for a 12-h incubation period before adding fluorescently dyed PLGA nanoparticles (approximately 250 nm in diameter) in excess. In order to add consistent NP concentration across experiments, NPs were pelleted, weighed and diluted to the same density, and then added at 0.001 mg/ml to each well. Uptake was allowed to occur for only 1 h, after which, cells were washed in PBS, lifted with scraping and analyzed by flow cytometry as previously described. Supernatant was again saved for cytokine analysis via ELISA. Four replicate wells were combined for each condition for each *N* and the experiment was repeated with multiple mice (*n* = 3).

### 3T3 Fibroblast Culture and the Induction of Apoptosis

NIH/3T3 (ATCC CRL-1658) mouse embryonic fibroblast line was first grown to confluence in DMEM complete media [10% FBS, 100 μg/ml (1%) penicillin/streptomycin/glutamine] on polystyrene tissue culture flasks. Fibroblast cells were then lifted and counted, and 3 million 3T3 fibroblasts were dyed by incubating 5 μM of Vybrant cell labeling solution DiO (benzoxazolium) with the fibroblasts at 37°C for 30 min. These fluorescently dyed cells were then resuspended in 5 mL of fresh warmed DMEM complete and seeded on polystyrene cell culture dishes. An additional 5 mL of warmed DMEM complete was then added to the culture dish and cells were allowed to seed for 12 h before being stimulated with 1 μg/ml ionomycin to induce apoptosis. Cell were incubated for an additional 12 h with ionomycin, to ensure complete apoptosis, before being lifted, spun down and then resuspended in 1 mL of warmed D10. 10 μl (∼35,000 apoptotic cells) of this apoptotic cell solution was then incubated with previously seeded BMDMs for 1 h of uptake.

### Apoptotic Cell Uptake

**FIGURE 3:**
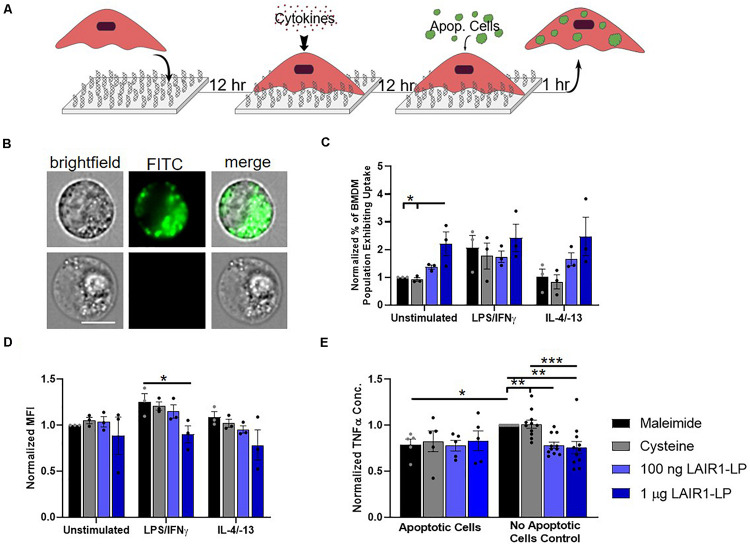
Effects of LAIR1-LP-conjugated surfaces on the uptake of apoptotic cells by BMDMs. **(A)** Diagram depicting experimental conditions and timeline used to investigate LAIR1-LP modulated uptake of apoptotic cells. **(B)** Representative flow cytometry microscopy images are shown of unstimulated BMDMs grown on maleimide surfaces (control) and incubated with DiO-labeled apoptotic 3T3 fibroblasts. Columns, from left to right, are images in brightfield, FITC wavelength (DiO), and combined brightfield/DiO (scale bar = 10 μm). **(C)** Percentage of BMDM cells that exhibited uptake of DiO-labeled apoptotic 3T3 fibroblasts on the different surfaces. Values have been normalized to the unstimulated BMDMs on maleimide. This value is representative of the propensity of the BMDM population for uptake on the different surfaces. **(D)** Mean fluorescence intensity of macrophages that exhibited uptake of DiO-labeled apoptotic cells. This metric is representative of the average amount of apoptotic cells taken up per BMDM (of the uptake positive BMDM population). Values have been normalized to unstimulated BMDMs on maleimide surfaces. **(E)** Normalized TNFα concentrations secreted by BMDMs that were stimulated with LPS/IFNγ, on the different surfaces, with and without apoptotic cells. For panels **(C–E)**, values reported are average ± S.E.M., with *N* = 3 independent biological replicates, and the dots are individual data points. Statistical significance was determined by one-way ANOVA with Tukey’s *post hoc* test (*p < 0.05, **p < 0.01, ***p < 0.001).

Bone marrow derived macrophages were seeded onto experimental surfaces (maleimide, cysteine, as well as unsaturated and saturated LAIR1-LP surfaces) at ∼30,000 BMDMs/well as previously discussed (Figure 3A). BMDMs were seeded for 12 h, and cells were stimulated with their respective stimulation solutions as previously mentioned and incubated for an additional 12 h. Apoptotic 3T3s were seeded for 12 h, lifted, and resuspended thoroughly in D10 media at a concentration of ∼3 million cells/ml (based on seeding density). BMDMs were co-incubated with ∼35,0000 apoptotic 3T3 fibroblasts and allowed to incubate for 1 h before being washed with PBS and lifted for analysis by flow cytometry. TNFα in the supernatant was analyzed by ELISA. Four replicate wells were combined for each condition for each *N* and the experiment was independently repeated (*n* ≥ 3 mice). For this experimental system apoptotic cells were not introduced in great excess since higher apoptotic cell to BMDM ratios caused aggregation and heterogeneous coverage of the surfaces.

### PLGA MP Uptake by LAIR-1 Knock-Down BMDMs

Knockdown of LAIR-1 gene was performed by nucleofection (4D-Nucleofector system, Lonza) using siRNAs (siGENOME siRNAs, Dharmacon). Briefly, 0.5 × 10^6^ freshly isolated BMDMs were transfected with 100 nM of siRNA in 20 μl of nucleofection solution. After nucleofection, cells were recovered in D-10 media (1X DMEM, 10% heat-inactivated FBS, 1% P/S, and 10% MCSF) and seeded on both 1 μg LAIR1-LP surfaces and maleimide control surfaces for 36 h and then co-stimulated with both cytokines (as previously described) and PLGA MPs (∼150,000 microparticles/well). The supernatant was collected 12 h post-stimulation and analyzed for cytokine secretion by enzyme-linked immunosorbent assay (ELISA) following manufacturer’s protocol (Biolegend). Cells were lifted and particle uptake was analyzed by flow cytometry.

Extent of LAIR-1 knockdown as well as LAIR-1 expression on the various surfaces was determined via qRT-PCR gene expression analysis. RNA was isolated from the BMDMs using TRI Reagent (Sigma T9424) following the manufacturer’s protocol. Briefly, cells were lysed directly on the culture dish using TRI Reagent, and chloroform was to the lysate. The samples were vortexed vigorously for 15 s and allowed to stand at RT for 10 min. Then, the mixture was centrifuged at 12,000 *g* for 15 min at 4°C. The upper aqueous phase that contains RNA was separated carefully to a new tube and 0.5 ml of 2-propanol per ml of TRI Reagent used was added. Then the samples were allowed to stand at RT for 10 min and further centrifuged at 12,000 *g* for 10 min at 4°C. The RNA pellet was washed with 1 ml of 75% ethanol per 1 ml of TRI Reagent used in sample preparation. The pellet was briefly air-dried, and the RNA was dissolved in DEPC-treated water.

The cDNA was made using High Capacity cDNA Reverse Transcription Kit (Applied Biosystems). 1 μg of total RNA was used to synthesize cDNA using random primers provided in the kit following the manufacturer’s protocol. Once the cDNA was made, it was diluted 10 times with ultrapure water and used for qPCR. Quantabio’s PerfeCTa SYBR Green FastMix was used for real-time PCR. Briefly, for every 25 μl reaction, 12.5 μl of the PerfecCTa SYBR Green Fastmix, 2 μl of diluted cDNA and 1 μl (500 nM) of each forward and reverse primers and 8.5 μl of ultrapure water was used. A total of 40 cycles were performed on Bio-Rad’s CFX-96 real-time PCR system. Further the relative gene expression was analyzed by 2^–ΔΔCT^ method (Livak and Schmittgen, 2001). Sequences of the siRNAs used for knockdown are presented in Supplementary Table SI-1.

### Flow Cytometry

Cell supernatant was removed, then wells were washed with 75 μl of PBS (1×), scraped, repeatedly pipetted to further lift cells, and then four repeat wells of ∼30,000 BMDMs in 75 μl of PBS were combined for quantitative analysis. A BD LSRII flow cytometer cell analyzer was used to determine the extent of uptake. All uptake materials were labeled using fluorescent dyes BODIPY (excitation 503 nm; emission 512 nm) or DiO (excitation 484 nm; emission 501 nm).

### Enzyme-Linked Immunosorbent Assay (ELISA)

At 12 h after stimulation, cell supernatant was collected and tumor necrosis factor alpha (TNFα) secretion levels were assessed by enzyme-linked immunosorbent assay (ELISA) following manufacturer’s protocol (BioLegend).

### ImageStream Flow Cytometry

Cell supernatant was removed, then wells were washed with 75 μl of PBS (1×), scraped with a cell scraper, repeatedly pipetted to further lift cells, and then four repeat wells of ∼30,000 BMDMs in 75 μl of PBS were combined, then spun down and resuspended in 20 μl of PBS for qualitative analysis. Amnis ImageStream Mark II Imaging Flow Cytometer was used to image individual BMDMs to visualize the extent of cellular uptake. Images were sorted using internalization software/application of the Amnis ImageStream Mark II Imaging Flow Cytometer.

### Statistical Analyses

Statistical significance for macrophage experiments was determined using a one-way ANOVA test with Tukey’s *post hoc* multiple comparisons test analysis via Prism 8 software, unless otherwise described in the figure caption. *P*-values of *p* < 0.05 were considered to be statistically significant. Any normalized data are stated in each figure caption. All experiments were repeated independently at least 3 times (*n* ≥ 3) and values were represented as the mean ± the standard error of the mean (S.E.M.), unless otherwise indicated.

## Results

### PLGA Microparticle Phagocytosis

To study the effects of LAIR1-LP-functionalized surfaces on the uptake of microparticles (MPs), PLGA MPs were incubated with BMDMs on surfaces with and without LAIR1-LP following the schedule shown in Figure 1A. SEM images were taken of the PLGA MPs. Analysis of these images showed that MPs exhibited an average diameter of 2.5 μm ± 0.75 μm, with a PDI of 0.09 (Figure 1B). ImageStream analyses of images that were obtained at internal focal planes confirmed that the PLGA MPs were successfully taken up by BMDMs (Figure 1C), and >90% of the cells which were gated to have positive fluorescent signal showed MPs that were internalized by cells.

LAIR1-LP interaction with LAIR-1 requires a trimeric structure of the peptide (Brondijk et al., 2010) and we confirmed the triple-helical folding of the LAIR1-LP peptides by circular dichroism (Supplementary Figure SI-1). The amount of peptide conjugated to the surface and its relative surface saturation was examined by a streptavidin-HRP assay (Supplementary Figure SI-2). This data was used to determine the concentrations of LAIR1-LP to investigate.

At the time point examined (24 h), we found that almost all cells exhibited MP uptake across LAIR1-LP and control surfaces (Supplementary Figure SI-5). However, LAIR1-LP peptide-functionalized surfaces significantly increased the amount of PLGA MPs taken up by BMDMs to approximately two-fold in every stimulated phenotype (i.e., M0, M1, and M2), when compared to the effects on cysteine-functionalized (no LAIR1-LP) surfaces (Figure 1D). Although we recognize that the boundaries for classification of the macrophage phenotypes into M0 (resting, non-activated), M1 (classically activated, pro-inflammatory), and M2 (alternatively activated, pro-healing) are not strictly distinct, for simplicity we will describe BMDMs stimulated with LPS/IFNγ and with IL-4/IL-13 as M1 and M2 conditions, respectively.

As phagocytosis was increased, the LAIR1-LP functionalized surfaces simultaneously resulted in a significant reduction (∼40%) in the inflammatory cytokine TNFα by LPS/IFNγ-stimulated BMDMs, when compared to TNFα levels on the non-LAIR1-LP control surfaces (Figure 1E). The decrease in TNFα secretion on LAIR1-LP surfaces, without the presence of microparticles, is consistent with our prior studies (Kim et al., 2017). Our prior investigation had also showed that an irrelevant control peptide (e.g., an ovalbumin epitope) that was conjugated to the surface elicited similar low TNFα levels as those observed for maleimide and cysteine-conjugated control surfaces (Kim et al., 2017).

### PLGA Nanoparticle Uptake

To investigate the effects of LAIR1-LP functionalized surfaces on the uptake of nanoparticles (NPs), PLGA NPs were incubated with BMDMs and seeded on surfaces conjugated with and without LAIR1-LP (Figure 2A). Dynamic light scattering analysis determined that the average NP size was 214 nm in diameter with an average PDI of 0.035 (Supplementary Figure SI-3), and TEM images confirmed the shape and size of PLGA NPs (Figure 2B). ImageStream analysis qualitatively confirmed that the PLGA NPs were successfully taken up by BMDMs (Figure 2C).

We observed again that approximately 90% of BMDM on all surfaces exhibited NP uptake at the time point examined (1 h) (Supplementary Figure SI-5), but that unstimulated BMDMs (M0) seeded on the LAIR1-LP saturated surfaces (1 μg/well) exhibited a significant (∼25%) increase in the average total uptake of PLGA NPs per BMDM when compared to both control surfaces without LAIR1-LP (*p* < 0.05; Figure 2D). Although the M1 and M2 conditions did not show statistically significant uptake differences between the macrophages grown on the LAIR1-LP vs. no-LAIR-LP surfaces, the trends were similar to those observed for the MPs, and the averages in uptake were higher on LAIR1-LP surfaces. Unlike the results for the MPs, there were no significant differences in the pro-inflammatory TNFα secretion between M1-stimulated macrophages which had taken up nanoparticles and were cultured on the different surfaces, although the trends did show a decrease in the average TNFα concentration on LAIR1-LP surfaces (Figure 2E). Therefore, while increases in MP uptake is correlated with decreased TNFα secretion on LAIR-LP conjugated surfaces, the relationship between uptake and decreases in TNFα concentrations appear to be less significant for NPs.

### Apoptotic 3T3 Fibroblast Uptake

Macrophages clear apoptotic cells as part of their natural biological processes, so we studied the effects of LAIR1-LP functionalized surfaces on the uptake of apoptotic bodies (Figure 3A). ImageStream images that qualitatively depict the uptake of apoptotic fibroblasts by BMDMs are presented in Figure 3B. Internalization analysis determined that >99% of the cells gated to have positive fluorescent signal showed complete internalization of apoptotic cells by BMDMs. Due to the tendency of apoptotic bodies to aggregate at high concentrations and lead to heterogenous coverage of BMDMs, the “saturated population” uptake condition that we obtained for MP and NP studies (Supplementary Figure SI-5) could not be achieved. Saturated uptake is defined as the average percentage of unstimulated BMDMs exhibiting uptake is ≥80% on all surfaces. Therefore, an additional metric describing the percentage of macrophage population exhibiting uptake (Figure 3C) was used in combination with MFI (Figure 3D) to more completely describe the effects of LAIR1-LP on macrophage uptake of apoptotic cells.

Our data demonstrated LAIR1-LP’s ability to significantly increase the percentage of unstimulated BMDMs exhibiting uptake of apoptotic 3T3 cells by greater than twice the values obtained on either of the control surfaces (Figure 3C). M2 BMDMs (with IL-4/IL-13) also showed this increasing trend, but the increase was just under statistical significance. These LAIR1-LP-induced increases in percentage of BMDMs exhibiting uptake were also conserved for PLGA MPs at unsaturated conditions (Supplementary Figure SI-6). Interestingly, although LAIR1-LP surfaces had no effect on the percentage of M1 (LPS/IFNγ-stimulated) BMDMs exhibiting uptake, the ligand peptide exerted an inhibitory effect on the average total uptake of apoptotic 3T3 fibroblasts by M1-stimulated BMDMs, with a significant ∼30% arrest of uptake when compared to uptake on maleimide surfaces (Figure 3D). This trend of decreased average uptake on LAIR1-LP surfaces was also observed for M0 and M2 phenotypes, but was not determined to be statistically significant.

We found that apoptotic cells had an anti-inflammatory effect on M1-stimulated BMDMs. The macrophage TNFα secretion response to apoptotic cells were equivalent on every surface, and were at comparable levels as the TNFα of BMDMs seeded on LAIR1-LP surfaces but with no apoptotic cells (Figure 3E). We observed a significant ∼20% decrease in TNFα secretion between the BMDMs on maleimide and cysteine control surfaces, when apoptotic cells were present (*p* < 0.05). Therefore, any potential anti-inflammatory effects of LAIR1-LP could not be discerned against the anti-inflammatory background of the apoptotic bodies. The anti-inflammatory effects of apoptotic bodies exemplified in this study are well corroborated by previous research (Fadok et al., 1998).

### Knockdown (KD) of LAIR-1 Expression in BMDM and Its Effect on Phagocytosis of PLGA MPs

To examine the contribution of LAIR-1 on our observed increase in phagocytosis on LAIR1-LP surfaces, we examined the effects of knocking-down LAIR-1 gene expression. Baseline expression, without gene knock-down, was first determined. Gene expression of LAIR-1 by BMDMs under each stimulation condition (M0, M1, and M2), seeded on the LAIR1-LP and control surfaces, indicated that the expression of LAIR-1 was affected by surface material (Supplementary Figure SI-4). Gene expression differences on the control surfaces (maleimide- and cysteine-functionalized) among the M0, M1, and M2 phenotypes were not observed to be statistically different, although M1 macrophages showed approximately half the average LAIR-1 gene expression of M0 and M2 macrophages on maleimide surfaces, and on cysteine-functionalized surfaces LAIR-1 expression on M1 and M2 macrophages were increased relative to M0 values. In contrast, on LAIR1-LP surfaces, LAIR-1 expression was doubled in M2 phenotypes relative to M0 macrophages (*p* < 0.05).

**FIGURE 4:**
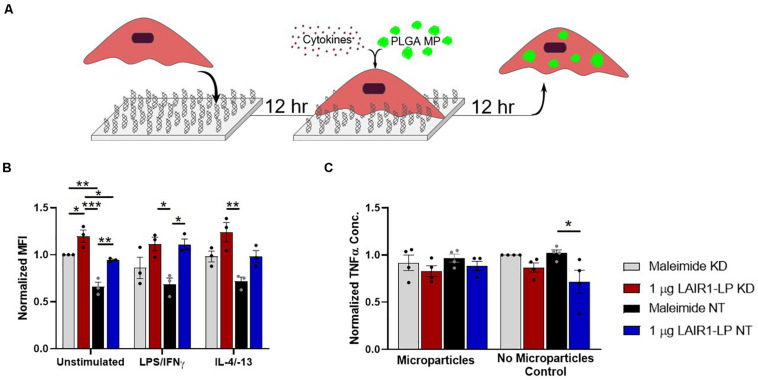
Effects of LAIR-1 expression on LAIR1-LP-conjugated surface-mediated uptake of PLGA MPs by BMDMs. **(A)** Diagram depicting the experimental conditions and experimental timeline used to investigate the effects of LAIR-1 expression on uptake of PLGA MP. **(B)** Mean fluorescence intensity of BODIPY-labeled PLGA MPs, in macrophages with internalized particles. LAIR-1 expression was decreased by knockdown (KD) of the LAIR-1 gene, compared to the non-targeted (NT) control. Values were normalized to unstimulated control KD BMDMs on maleimide surfaces. Values reported are average ± SEM, with *N* = 3 independent biological replicates, and a two-tailed unpaired *t*-test was used (*p < 0.05, **p < 0.01, ***p < 0.001). **(C)** Normalized ELISA data depicting the average TNFα concentration of LPS/IFNγ stimulated BMDMs (with LAIR-1 KD and NT) on surfaces with and without LAIR1-LP, incubated with and without PLGA MPs. Values reported are average ± S.E.M., with *N* = 3 independent biological replicates, and the dots are individual data points. Statistical significance was determined by one-way ANOVA with Tukey’s *post hoc* test (*p < 0.05, **p < 0.01, ***p < 0.001).

LAIR-1 knockdown (KD) was effective and decreased LAIR-1 gene expression by 90% when compared to the non-target (NT) control (Supplementary Figure SI-7). Uptake from these LAIR-1 KD cells were evaluated according to the MP uptake protocol (Figures 1A, 4A). LAIR-1 knockdown of unstimulated (M0) macrophages increased the average amount of PLGA MPs phagocytosed per BMDM on both maleimide (control) and LAIR1-LP-conjugated surfaces; in particular, the KD of LAIR-1 expression resulted in a significant ∼35% increase in MFI on non-peptide surfaces, and a ∼25% increase in uptake in cells cultured on LAIR1-LP surfaces (Figure 4B). The results also showed that LAIR1-LP induced significant increases of ∼20 and ∼30% in uptake per cell for both LAIR-1 KD and non-target (NT) BMDMs, respectively. The trends for all groups of both M1 and M2 phenotypes also supported the significant increases observed in the unstimulated M0 macrophages (Figure 4B).

Decreasing the LAIR-1 gene expression did not significantly decrease the level of TNFα on LAIR1-LP surfaces for M1 (LPS/IFNγ) macrophages that were incubated with or without MPs (Figure 4C). However, the LAIR1-LP surface-induced decrease in TNFα (relative to the maleimide surface) that had previously been observed (Figure 1E) was conserved for the NT control group. The previous LAIR1-LP-induced decrease in TNFα during MP phagocytosis (Figure 1E) was not observed in either the LAIR-1 KD or NT BMDMs (Figure 4C).

**FIGURE 5:**
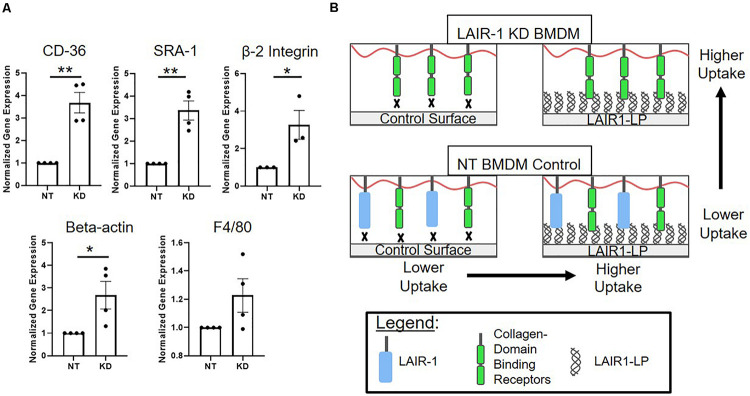
**(A)** Relative gene expression of receptors involved in uptake. Cd36, SRA-1(Msr-1), and beta 2 integrin (Itgb2), were analyzed via qPCR, along with structural protein beta-actin (Actb) and F4/80 (Adgre1) which were used as controls. Gene expression is normalized to non-targeted (NT) polystyrene condition. Data presented as mean ± S.E.M. of 3 or more independent biological replicates, and the dots are individual data points. *p < 0.05, **p < 0.01, ***p < 0.001, assessed by two-tailed Student’s *t*-test. **(B)** Diagram depicting a proposed model consistent with gene expression data from LAIR-1 KD (top) and NT (bottom) BMDMs, and cultured on control surfaces (left) and LAIR1-LP functionalized surfaces (right).

Interestingly, we observed that when LAIR-1 gene expression was decreased by KD, the expression of other receptors and molecules that have been implicated in cell uptake increased. Gene expression of CD36, SRA-1, integrin β2, and β-actin were examined, and all were significantly increased (Figure 5A). It has been shown in previously published studies that gene and protein expression of LAIR-1, CD-36, and SRA-1 are positively correlated (Zibara et al., 2002; Yi et al., 2018). F4/80, a surface marker for macrophages but is not known to be linked to uptake, was the control and showed no increase in expression.

## Discussion

To systematically characterize the immunomodulatory effects of LAIR1-LP on macrophage uptake, we conducted *in vitro* studies utilizing LAIR1-LP functionalized surfaces, bone marrow derived macrophages, and a variety of uptake materials. Our data show that the engineered collagen peptide, LAIR1-LP, has a complex immunomodulatory effect, increasing cellular uptake while decreasing inflammatory response. We also demonstrate, through knockdown studies, the inhibitory effects of LAIR-1 on macrophage uptake and TNFα production.

We observed that LAIR1-LP surfaces significantly increased the average PLGA MP phagocytosis per BMDM across the M0, M1, and M2 phenotypes and decreased the overall inflammatory response of BMDMs in LPS/INFγ-stimulated condition, when compared to control surfaces (Figures 1D,E). PLGA is a biodegradable polymer and has been used in several FDA-approved medical devices, and has also been examined for its drug loading capabilities and its potential to improve surface to host cell interactions (Sun et al., 2017). PLGA MP uptake is relevant to drug delivery as PLGA has been used as vehicles for hydrophobic drug payloads (Abdelaziz et al., 2018; Liu et al., 2018). In the context of implant integration, PLGA MPs can be a model for debris from PLGA-based medical devices/implants (Yoshida and Babensee, 2006; Yang et al., 2009), which are often plagued with inflammation (Kim and Park, 2006; Jiao and Cui, 2007; Brandhonneur et al., 2009; Sun et al., 2017; Rowley et al., 2019). Our PLGA uptake data suggests the potential of integrating LAIR1-LP into materials as a possible strategy to improve biomaterials for drug delivery and medical devices/implants. Engineering LAIR1-LP functionalized hydrogels could be particularly useful in treating inflammatory diseases, such as arthritis. Since LAIR1-LP surfaces increase PLGA MP macrophage uptake while decreasing their TNFα production, engineering PLGA implant coatings with LAIR1-LP may improve implant integration by decreasing the foreign body response while increasing the clearance of degraded PLGA debris.

Poly(lactic-co-glycolic acid) particles are also relevant on a nanoscopic scale in drug delivery (Bobo et al., 2016; Abdelaziz et al., 2018; Liu et al., 2018; Skorobogatova et al., 2019), and therefore we also examined the effects of LAIR1-LP on PLGA NP uptake. The experimental data showed the trend of LAIR1-LP surfaces increasing the average amount of PLGA NP taken up per BMDM when compared to control surfaces (Figure 2D). Similar to the MP data, BMDMs stimulated by inflammatory condition (LPS/IFNγ) on LAIR1-LP surfaces and incubated with NPs exhibited trends of decreasing TNFα, although these trends were not statistically significant (Figure 2E). The effects of LAIR1-LP on PLGA NP uptake was less impactful than its effects on PLGA MP phagocytosis. One reason may be that unlike NPs, the uptake of MPs is directed more significantly via surface receptors and cell membrane interactions, resulting in increased inflammatory response in macrophages when compared to nanoparticle uptake (Nicolete et al., 2011). This, together with increased activation by MPs (Figures 1E, 2E), may account for the more significant effects of LAIR1-LP when compared to PLGA NP uptake. Another possible reason for the larger differences within the MP groups, compared to the NP data, is the different experimental times required to achieve “saturated” uptake (Supplementary Figure SI-5); NP uptake happens quickly, which experimentally limited NP uptake to a 1-h incubation period, while MP uptake occurs more slowly (12 h). This could explain why similar trends were observed between NP and MP data, but the effects of LAIR1-LP may not have been as distinguishable, even after only 1 h of uptake, for the NPs.

Physiologically, macrophage uptake of apoptotic cells plays a critical role in maintaining homeostatic control in the body. The overall trends we observed in this study shows that LAIR1-LP surfaces increased the percentage of macrophage uptake of apoptotic bodies (Figure 3C) and decreased the average uptake of apoptotic bodies per macrophage (Figure 3D). Apoptotic bodies have been demonstrated to elicit anti-inflammatory effects on macrophages, with LAIR-1 being implicated in the mechanisms driving these results (Savill, 1997; Fadok et al., 1998; Son et al., 2016; Son et al., 2017; Thielens et al., 2017). Thus, additional anti-inflammatory effects of the peptide were not observed, and the expected LAIR1-LP-induced decreases in TNFα were not discernable in the presence of apoptotic bodies (Figure 3E). Apoptosis is a mechanism for programmed cell death that results in the surface display of normally internal proteins, which signals the immune system to initiate uptake. Besides uptake signal proteins, apoptotic cell surfaces have autoantigens, which induce an immune response that can be harmful to healthy tissue if they accumulate to high concentrations (Casciola-Rosen et al., 1994). Low apoptotic cell clearance has been linked to autoimmune diseases such as Systemic Lupus Erythematosus (SLE) (Mevorach et al., 1998). The results of our apoptotic uptake experiments suggest that LAIR1-LP may prime macrophages to increase clearance of apoptotic bodies. These observed effects motivate future study on the potential of LAIR1-LP and other collagen-mimetic peptides as a therapeutic biomaterial for the treatment of autoimmune diseases.

After observing the multifaceted immunomodulatory effects of the LAIR1-LP surfaces on BMDMs of different phenotypes, we examined how the expression of LAIR-1, the target surface receptor for LAIR1-LP-conjugated surfaces, played a role in the exhibited increases in macrophage uptake. The LAIR-1 KD data showed that the higher expression of LAIR-1 decreased the average amount of cellular uptake on both control and LAIR1-LP surfaces (Figure 4B). These findings are consistent with published literature showing that knockdown of LAIR-1 increased the uptake of lipids (Yi et al., 2018). However, knocking down LAIR-1 expression arrested the anti-inflammatory effects (TNFα levels) of LAIR1-LP on M1-stimulated BMDMs, which is consistent with previously published results (Kim et al., 2017).

One explanation for the KD-induced increase in uptake may be linked to our observation that decreasing the expression of LAIR-1 affected the expression of other receptors that are related to uptake; gene expression for CD36, SRA-1, and beta-2 integrin all significantly increased when LAIR-1 was knocked-down (Figure 5A). These apparent correlations suggest that the expression of surface receptor LAIR-1 may not be directly responsible for the increase in uptake observed on LAIR1-LP-coated surfaces, and that competitive binding of LAIR1-LP may exist between LAIR-1 and other collagen-recognizing surface receptors. Several cell surface receptors, such as beta-2 integrin, CD36, LRP-1, and CR3 bind to collagen and/or collagen-like domains and have been implicated in upregulation of various mechanisms of cellular uptake (Newman and Tucci, 1990; Herz and Strickland, 2001; Edelson et al., 2006; Lahti et al., 2013). A model, which is consistent with our observed data, for LAIR-1 and collagen-binding cell receptors that are involved in uptake is proposed in Figure 5B. Our gene expression data of CD36 and SRA-1 on LAIR1-LP surfaces do not show any increase as compared to cysteine control surfaces (Supplementary Figure SI-9). Based on literature and observed experimental results, it is likely that some of these collagen recognizing surface receptors involved in uptake may also bind LAIR1-LP. However, due to functional redundancy of the many receptors involved in uptake (Moore et al., 2005; Prapainop et al., 2017), knockdown of individual receptors may not completely prevent internalization. In contrast, since the actin cytoskeleton is involved in uptake through many receptors, it is possible that its inhibition would have a more pronounced effect (May and Machesky, 2001). Our data may suggest a correlation with non-collagen binding proteins that are associated with uptake as well; the expression of scavenger receptor SRA-1, a receptor critical in macrophage uptake (Yamada et al., 1998) and of beta-actin, which is involved in cell uptake, were also increased in the LAIR-1 KD macrophages. We speculate that the increased expression of SRA-1, beta-actin, and other non-collagen binding proteins associated with uptake in LAIR-1 KD BMDMs could explain the observed increase in uptake by LAIR-1 KD BMDMs on maleimide surfaces when compared to NT BMDMs (Figure 4B).

The dualistic nature of LAIR1-LP-induced response results in generally increasing macrophage uptake while simultaneously decreasing their inflammatory response. Integrating LAIR1-LP into novel biomaterials could elicit targeted immunomodulatory control, which would be ideal for a variety of medical and engineering applications. This research also highlights the possibilities for further investigations into the complex, multifaceted, and regulatory role of collagen and collagen-domains in directing innate immune responses.

## Data Availability Statement

The raw data supporting the conclusions of this article will be made available by the authors, without undue reservation, to any qualified researcher.

## Ethics Statement

The animal study was reviewed and approved by IACUC, University of California at Irvine.

## Author Contributions

AR, VM, EC, S-WW, and WL conceptualized and designed the experiments. AR, VM, and NW-W performed the experiments, wrote the manuscript, and prepared the figures. S-WW and WL reviewed and edited the manuscript. All authors contributed to the article and approved the submitted version.

## Conflict of Interest

The authors declare that the research was conducted in the absence of any commercial or financial relationships that could be construed as a potential conflict of interest.

## Abbreviations

BMDM: bone marrow derived macrophages
IFN γ: interferon gamma
IL-13: interleukin 13
IL-4: interleukin 4
KD: knockdown
LAIR-1: leukocyte-associated immunoglobulin-like receptor-1
LAIR1-LP: LAIR-1 ligand peptide (collagen-mimetic peptide)
LPS: lipopolysaccharide
MP: microparticles
NP: nanoparticles
NT: non-targeted
PLGA: poly(lactic-co-glycolic acid)
SRA-1: scavenger receptor class A – 1.
