# Supplementary material for: Effects of Surface-Bound Collagen-Mimetic Peptides on Macrophage Uptake and Immunomodulation
Source: Front Bioeng Biotechnol. 2020 Jul 3;8:747. doi: 10.3389/fbioe.2020.00747 (PMC7348040; doi:10.3389/fbioe.2020.00747)

Supplementary Information

Effects of surface-bound collagen-mimetic peptides on macrophage uptake and immunomodulation

Andrew T. Rowley,[1] Vijaykumar S. Meli,[1,2] Natalie Wu-Woods,[3] Esther Y. Chen,[1]

Wendy F. Liu,[1,2,4]* and Szu-Wen Wang[1,2]*

[1]Department of Chemical and Biomolecular Engineering

[2]Department of Biomedical Engineering

[3]Department of Materials Science and Engineering

[4]The Edwards Lifesciences Center for Advanced Cardiovascular Technology

University of California

Irvine, CA 92697 USA

Supplementary Methods

After nucleofection of BMDMs with LAIR-1 and non- target (NT) siRNAs, cells were cultured on tissue culture plastic for 48h. Then the cells were lysed, RNA was extracted and cDNA was made as described earlier. The expression of the surface receptors was analyzed by qPCR using primer described in Supplementary Table SI-1.

Table SI-1. Primers used to determine the expression of surface receptors

Gene	Primer sequence (5'-3')
**mLAIR1-1F**	TCCTCCTTTGTCTTTCCGCC
**mLARI1-1R**	CAGGAAGCCTGTCATCTGCA
**mC1qaF**	CACCAACCAGGAGAGTCCAT
**mC1qaR**	TCCTTTTCGATCCACACCTC
**(F4/80)mADGRE-F**	AACATGCAACCTGCCACAAC
**(F4/80)mADGRE-R**	TGAATTCCTGGAGCACTCATCC
**mCD36-F**	TCCTCTGACATTTGCAGGTCTATC
**mCD36-R**	AAAGGCATTGGCTGGAAGAA
**mSRA-1-F**	CATGAACGAGAGGATGCTGACT
**mSRA-1-R**	GGA AGG GATGCTGTCATTGAA
**(Beta-2)mITGB-F**	TCACCTTCCAGGTAAAGGTCAT
**(Beta-2)mITGB-R**	AGTTTTTCCCAATGTAGCCAGA
**mbActin-F**	TATGCCAACACAGTGCTGTC
**mbActin-R**	ACCGATCCACACAGAGTACTTG

Details of siRNA used:
Company name: Horizon (earlier called as Dharmacon)
SMARTpool: siGENOME Lair1 siRNA- cat no **M-057755-01-0005**
siGENOME Non-Targeting siRNA #2-cat no **D-001210-02-05**

Supplementary Figures

Figure SI-1. Circular dichroism of LAIR1-LP at a concentration of 0.18 mg/ml in 50 mM Acetic acid.

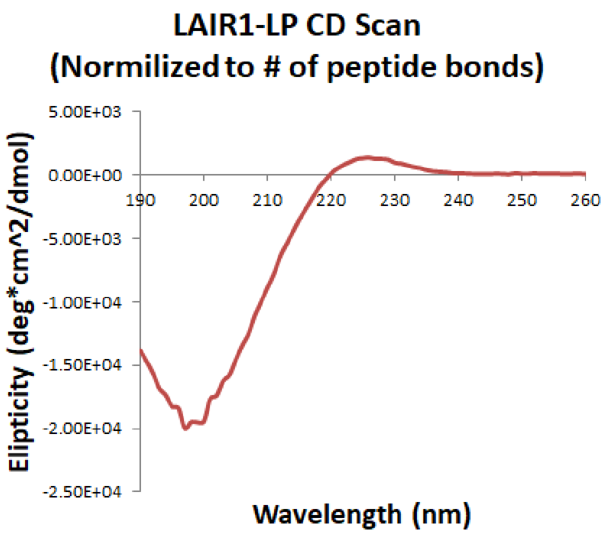

Figure SI-2. Surfaces functionalized with LAIR1-LP-Biotin reacted with FITC Streptavidin in 100x molar excess for one hour, before washing with PBS and analyzing with fluorescent plate reader.

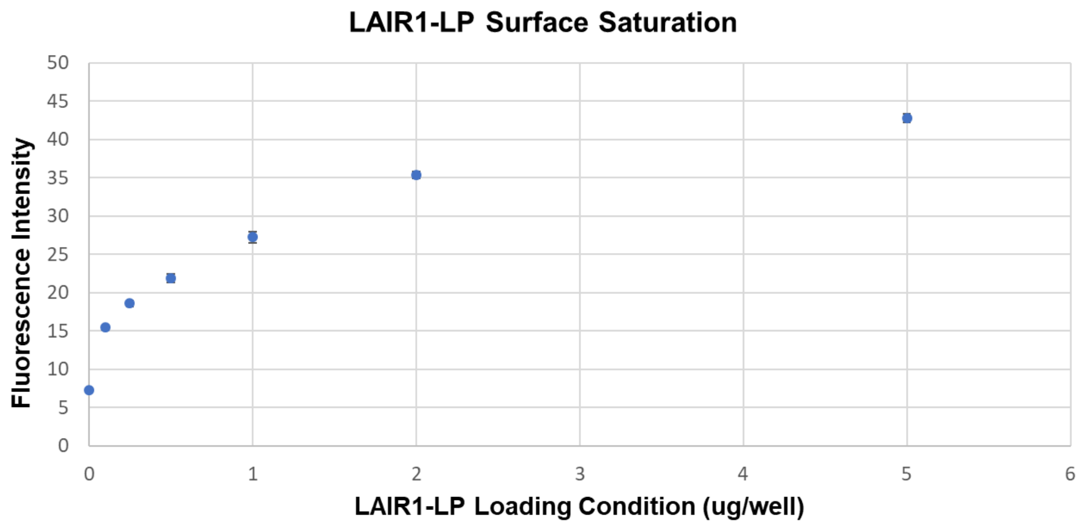

Figure SI-3. Representative Dynamic Light Scattering analysis showing the distribution of PLGA NP diameter. The average PLGA NP diameter was determined to be 214 ± 51 nm with an average PDI of 0.035 (n = 4).

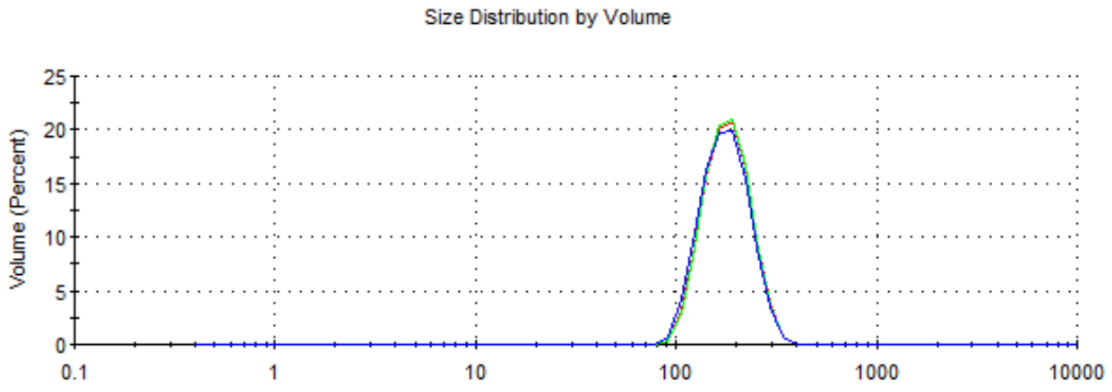

Figure SI-4. Gene expression data depicting the effects of experimental and control surfaces on LAIR-1 expression in the three predetermined phenotypes. Data is normalized to unstimulated BMDMs on each surface.

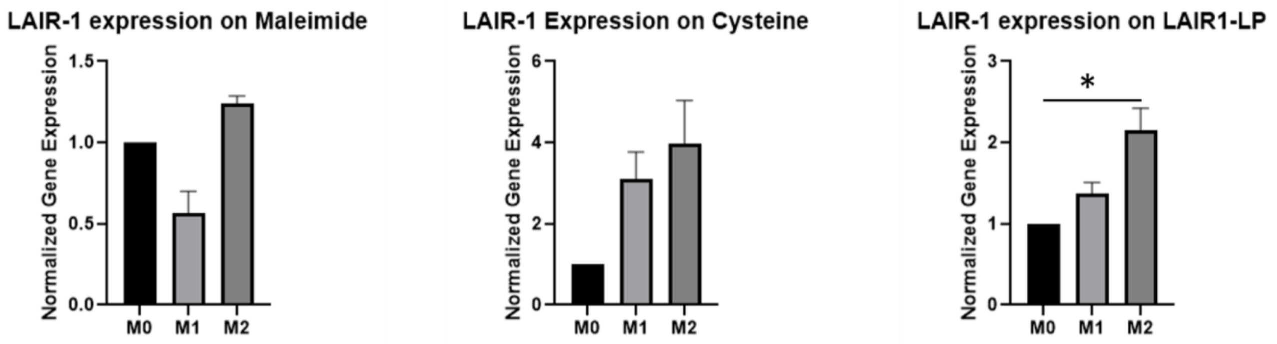

Figure SI-5. Percentage of BMDMs exhibiting "saturated" uptake of PLGA MPs after a 12-hour incubation period (top) and NPs after a 1-hour incubation period (bottom). Saturated condition is defined as the percentage of unstimulated BMDMs exhibiting uptake on average is greater than 80% on all surfaces.

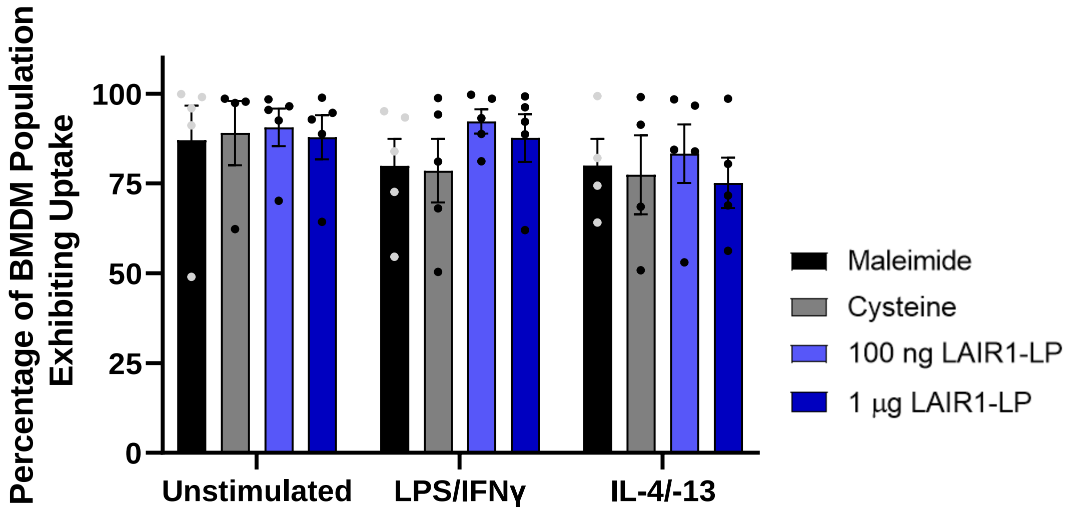

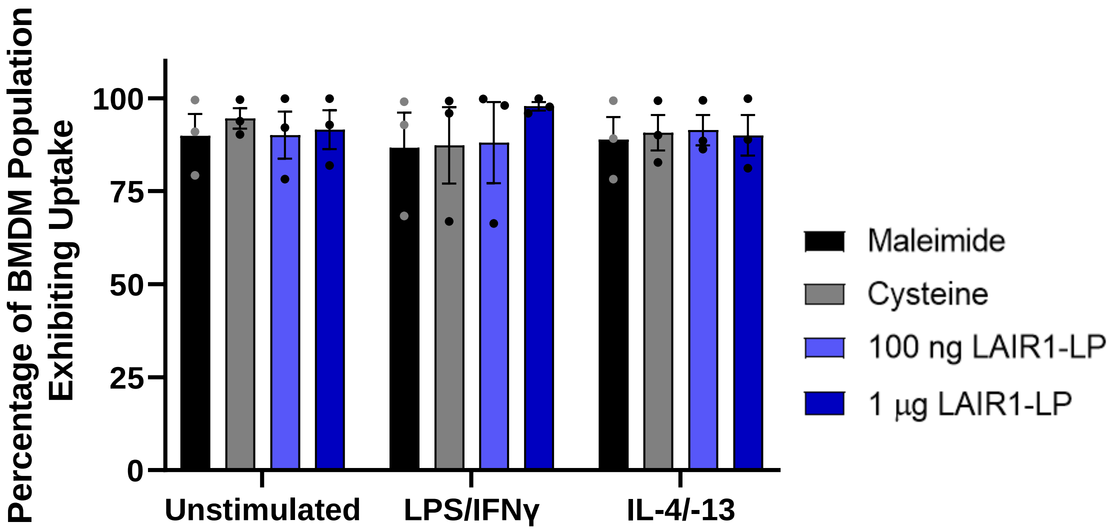

Figure SI-6. The effects of LAIR1-LP on the uptake of PLGA MPs, both percent populations exhibiting uptake and on average uptake per BMDM, at unsaturated conditions.

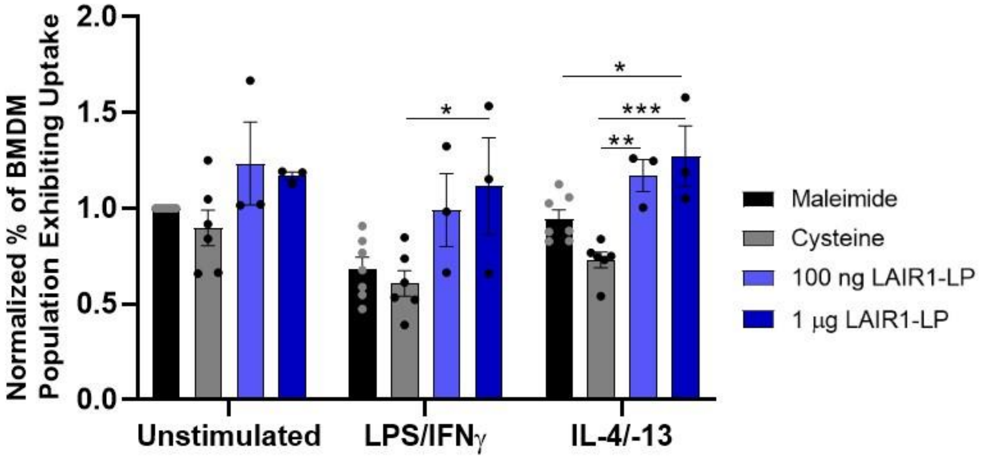

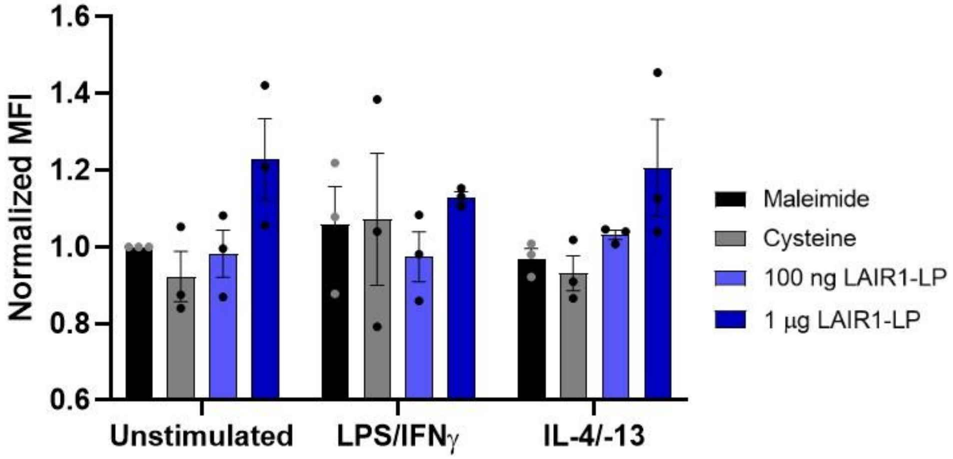

Figure SI-7. Gene expression displaying the extent of LAIR-1 knockdown.

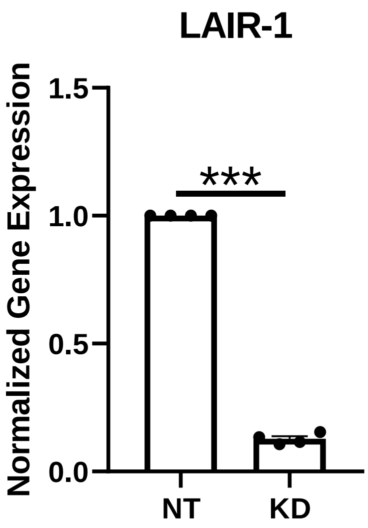

Figure SI-8. Representative flow cytometry dot plots depicting the raw data and active gates of PLGA MP uptake of LAIR-1 KD BMDMs. Showing both cell only and control group with positive uptake. (Top) M0 BMDMs on LAIR1-LP surface with PLGA MPs. (Bottom) M0 BMDM only control.

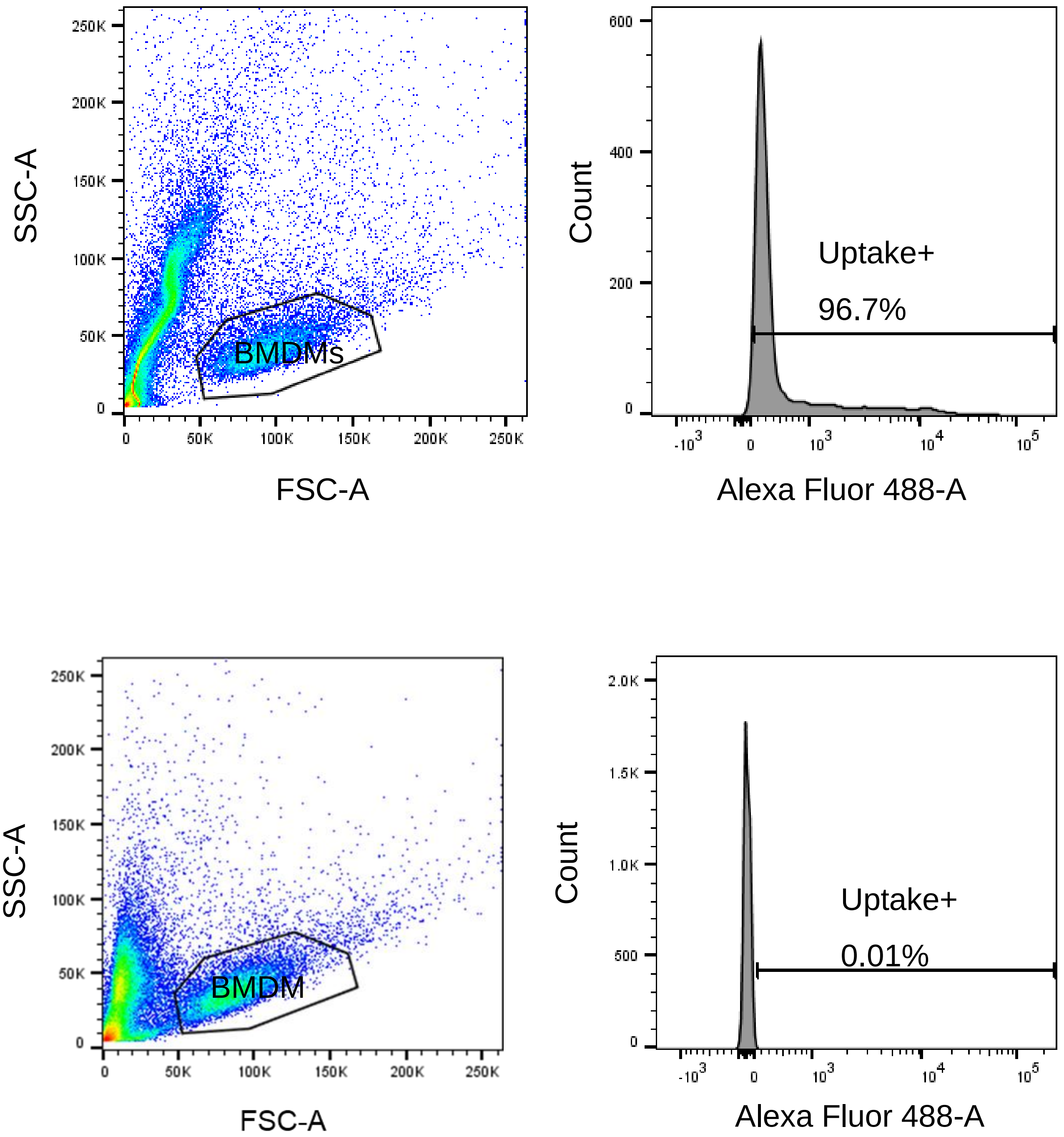

Figure SI-9: Gene expression showing CD36 and SRA-1 uptake receptor expression on the experimental surfaces. No significant gene expression differences between cells cultured on the cysteine and LAIR1-LP surfaces were observed.

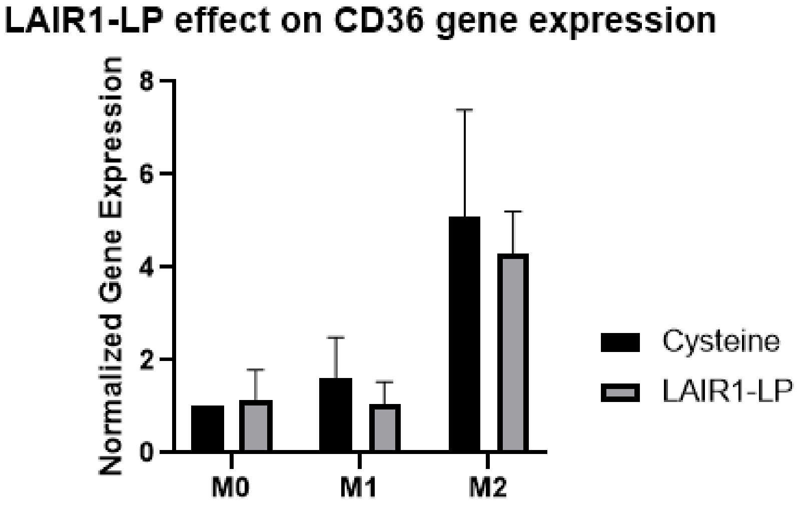

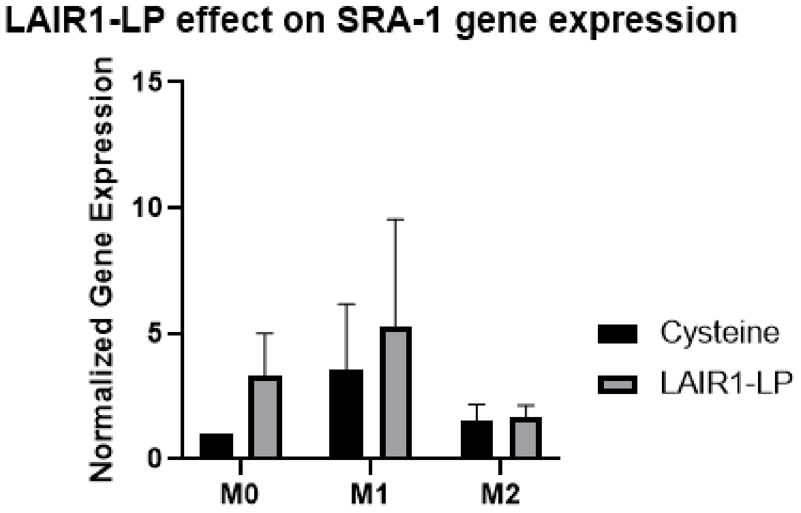

Supplement: Supplementary file 1 [file Data_Sheet_1.pdf]
